# Lack of a Clinically Meaningful Drug Interaction Between the HIV‐1 Antiretroviral Agents Islatravir, Dolutegravir, and Tenofovir Disoproxil Fumarate

**DOI:** 10.1002/cpdd.1026

**Published:** 2021-10-22

**Authors:** Deanne Jackson Rudd, Saijuan Zhang, Kerry L. Fillgrove, Sabrina Fox‐Bosetti, Randolph P. Matthews, Evan Friedman, Danielle Armas, S. Aubrey Stoch, Marian Iwamoto

**Affiliations:** ^1^ Merck & Co., Inc. Kenilworth New Jersey USA; ^2^ Celerion Tempe Arizona USA

**Keywords:** clinical pharmacology, clinical trials, drug‐drug interactions, HIV/AIDS, pharmacokinetics and drug metabolism

## Abstract

Islatravir, an investigational nucleoside reverse transcriptase translocation inhibitor, is in clinical development for the treatment and prevention of HIV‐1 infection. Because islatravir may be coadministered with other antiretroviral agents, assessment of potential drug‐drug interactions are warranted. This phase 1, open‐label, fixed‐sequence, 2‐period trial in adults without HIV (N = 12) assessed the safety and pharmacokinetic interactions of islatravir administered with dolutegravir and tenofovir disoproxil fumarate (TDF). In period 1, participants received a single oral dose of islatravir (20 mg). In period 2, participants received oral doses of dolutegravir (50 mg) and TDF (300 mg) once daily on days 1 through 11, with a single oral dose of islatravir (20 mg) coadministered on day 8. There were no clinically significant changes in islatravir, dolutegravir, or TDF pharmacokinetics following coadministration. Islatravir was generally well tolerated when administered alone or in combination with dolutegravir and TDF. Coadministration of islatravir, dolutegravir, and TDF is supported, with no clinically meaningful effect on pharmacokinetics, safety, or tolerability in participants without HIV.

The morbidity and mortality associated with HIV‐1 infection have been dramatically reduced with the advent of combination antiretroviral therapy,^1^ and HIV‐1 can now be managed as a chronic disease in which individuals can anticipate a near‐normal life expectancy.^1^, ^2^ Combination antiretroviral therapy typically involves the coadministration of therapeutic agents from different drug classes to suppress HIV‐1 replication.^3^ Despite the number of effective agents and combination regimens, current treatments are associated with limitations, such as adverse events and development of drug resistance.^3^, ^4^ As such, development of new and improved antiretroviral agents is warranted, including those that allow for alternative dosing schedules. Moreover, antiretrovirals with a low risk for drug interactions are warranted given the increasing need for polypharmacy among the aging population of people living with HIV‐1.^5^

Islatravir is an investigational nucleoside reverse transcriptase translocation inhibitor (NRTTI) in clinical development for the treatment and prevention of HIV‐1 infection.^6^, ^7^ Islatravir is rapidly converted by endogenous intracellular kinases to its pharmacologically active triphosphate form, which has demonstrated high potency against HIV‐1 and a long intracellular half‐life that allows for use of varying dosing schedules, from once daily to more extended durations.^8^, ^9^, ^10^ In vitro, islatravir inhibits multiple strains of HIV‐1, including common nucleoside reverse transcriptase inhibitor (NRTI)‐resistant variants, and may have a high barrier to the development of resistance.^9^, ^10^, ^11^ Investigations determined limited drug‐drug interactions of islatravir with no inhibitory effect on cytochrome p450 (CYP) biotransformation isoforms CYP1A2, 2B6, 2C8, 2C9, 2C19, and 2D6 at concentrations up to 100 μM and CYP3A4 up to 200 μM. No inhibitory effects were observed on uridine diphosphate glucuronosyltransferase 1A1 at concentrations up to 100 μM. Islatravir was not found to interfere with commonly associated drug transporters, pumps, transfer proteins, extrusion proteins, or resistance proteins and also displayed low levels of plasma protein binding. Existing evidence suggests that islatravir is unlikely to interact with major metabolic and transport pathways associated with commonly coprescribed medications.^12^

When administered orally as a single dose as low as 0.5 mg, islatravir demonstrated effective viral suppression for up to 7 days in treatment‐naive adults with HIV‐1.^13^ In adults without HIV, single oral doses of islatravir were rapidly absorbed, reaching peak plasma concentrations at ≈0.5 hours, with a plasma half‐life of ≈49 to 61 hours. Intracellular islatravir triphosphate levels peaked between 6 and 24 hours and demonstrated a half‐life of ≈118 to 171 hours.^14^, ^15^ The pharmacokinetic profile of islatravir was comparable in individuals with and those without HIV‐1. Islatravir has also been generally well tolerated across all clinical trials conducted to date.^13^, ^16^

Islatravir is being evaluated in combination with doravirine, a nonnucleoside reverse transcriptase inhibitor, as a once‐daily oral regimen for the treatment of HIV‐1. A phase 2b clinical trial demonstrated that islatravir in combination with doravirine was generally well tolerated and maintained virologic suppression for up to 48 weeks in treatment‐naive adults with HIV‐1; participants switched to the 2‐drug combination following initiation on a 3‐drug regimen of islatravir, doravirine, and lamivudine after 24 weeks.^17^ A comprehensive phase 3 clinical trial program to investigate the treatment potential of islatravir and doravirine across populations of people living with HIV‐1 (NCT04223778, NCT04223791, NCT04233879) will also include a trial in heavily treatment‐experienced individuals who are failing their current antiretroviral therapy regimen, in whom islatravir and doravirine will be coadministered in combination with optimized background antiretroviral therapy (NCT04233216). Because islatravir may be coadministered with other antiretroviral agents, assessment of the drug interaction with commonly used agents is warranted.

Dolutegravir, an integrase strand transfer inhibitor, and tenofovir disoproxil fumarate (TDF), an NRTI, are commonly used in combination and have no significant drug interactions with each other.^18^, ^19^ Dolutegravir‐based regimens are among the preferred first‐line regimens recommended in HIV‐1 treatment guidelines.^19^ Peak fasting plasma concentrations of dolutegravir are observed 0.5 to 1.25 hours following oral administration, with steady‐state concentrations achieved within ≈5 days following once‐daily multiple dosing.^20^ Peak plasma concentrations of tenofovir, the pharmacologically active form of TDF, are observed ≈1 hour after oral administration of TDF,^21^ with steady‐state concentrations achieved within ≈7 days following once‐daily dosing.^22^

A clinical trial was conducted to assess the safety and pharmacokinetic interaction between islatravir and the combination of dolutegravir and TDF in adult participants without HIV.

## Methods

### Investigational Review Board

The trial protocol, protocol amendment, and informed consent forms were approved by the Chesapeake Research Review (Columbia, Maryland) institutional review board. Before initiation of the trial, participants provided written informed consent to the procedures of the trial, with acknowledgment of the potential risks. The trial was conducted at Celerion (Tempe, Arizona) in conformance with Good Clinical Practice and following applicable local requirements regarding ethical committee review, informed consent, and other statutes or regulations regarding the protection of the rights and welfare of humans participating in biomedical research.

### Trial Design

**Figure 1:**
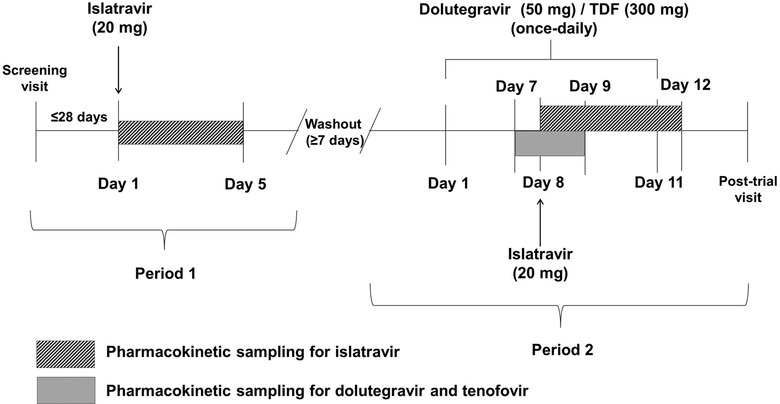
Study design. TDF, tenofovir disoproxil fumarate.

This was a phase 1, open‐label, fixed‐sequence, 2‐period trial in adults without HIV (protocol MK‐8591‐005). In period 1, participants received a single oral dose of 20 mg islatravir. In period 2, participants received multiple oral doses of 50 mg dolutegravir and 300 mg TDF once daily for 11 consecutive days (days 1‐11), with a single oral dose of 20 mg islatravir coadministered on day 8. There was a washout period of ≥7 days between islatravir dosing in period 1 and the first dose of dolutegravir and TDF in period 2 (Figure 1).

Dose selection for islatravir in this trial was based on preliminary data obtained from a monotherapy efficacy trial in treatment‐naive adults with HIV‐1.^23^ Based on a pharmacokinetic threshold that is projected to achieve intracellular concentrations of active islatravir triphosphate at efficacious levels, a dose of 20 mg islatravir was selected. A dose of 20 mg is anticipated to represent the upper range for treatment with weekly dosing and achieves islatravir triphosphate levels similar to those achieved at steady state with 0.75 mg daily dosing. For dolutegravir and TDF, the dose selected is the current US Food and Drug Administration–approved marketed dose as per labeling.

### Participants

Eligible participants were male or female adults aged 18 to 55 years, of nonchildbearing potential, and with a body mass index of 19 to 32 kg/m^2^. Key exclusion criteria included preexisting health conditions determined by the investigator to be clinically significant; infection with HIV, hepatitis B virus, or hepatitis C virus; nicotine or illicit drug use; creatinine clearance <80 mL/min; or an inability to refrain from the use of any medication, including prescription and nonprescription and herbal remedies.

### Assessments

#### Pharmacokinetics

Blood samples for islatravir plasma analysis were collected before dosing and at selected time points over 96 hours following administration of islatravir on day 1 of period 1 and day 8 of period 2. In period 2, samples taken at 24, 48, and 72 hours for islatravir plasma analysis were collected before dose administration of dolutegravir and TDF. Blood samples for plasma analysis of dolutegravir and tenofovir were collected before dosing on days 1, 7, and 8 of period 2 and at selected time points over 24 hours after dosing on days 7 and 8 of period 2. The 24 hour samples for dolutegravir and tenofovir analysis for days 7 and 8 were collected before dosing of dolutegravir and TDF.

The following pharmacokinetic parameters were assessed as appropriate for each drug: area under the plasma concentration–time curve from 0 to infinity (AUC_0‐inf_), area under the plasma concentration–time curve over the last 24‐hour dosing interval (AUC_0‐24_), maximum observed plasma concentration (C_max_), plasma concentration at 24 hours after dosing (C_24_), time to reach C_max_ (t_max_), and apparent terminal half‐life (t_1/2_).

Plasma concentrations of islatravir, dolutegravir, and tenofovir were determined by inVentiv Health Clinique (Québec City, Québec, Canada) using validated high‐performance liquid chromatography coupled to tandem mass spectrometry methods. All values below the lower limit of quantitation were treated as 0.

Islatravir (m/z 294.000/154.000) was extracted using an automated protein precipitation method using [^13^C,^15^N_3_]‐ISL as internal standard (m/z 297.800/158.000). The extracted samples were assessed on a system equipped with a X Select HSS T3 C18, 50×2.1 mm, 2.5 μm column with water/acetonitrile with formic acid mobile phase. Over the calibration range, intraday precision and accuracy ranged from 3.38% to 4.21% coefficient of variation (CV) and 101.23% to 104.57%, respectively. Interday precision and accuracy ranged from 1.20% to 4.61% CV and 87.79% to 106.87%, respectively. The lower limit of quantitation for islatravir was 0.10 ng/mL, with an analytical range of 0.10 to 100.00 ng/mL.

Dolutegravir (m/z 420.200/277.200) was extracted using an automated protein precipitation method using [C^13^‐d_5_]‐dolutegravir as the internal standard (m/z 426.300/277.300). The extracted samples were assessed on a system equipped with an ACE 3 C18, 30 × 4.6 mm, 3 μm column (Avantor, Radnor, Pennsylvania) with water/methanol with ammonium formate and formic acid mobile phase. Over the calibration range, intraday precision and accuracy ranged from 0.91% to 2.50% CV and 99.44% to 103.82%, respectively. Interday precision and accuracy ranged from 0.16% to 19.80% CV and 99.96% to 109.18%, respectively. The lower limit of quantitation for dolutegravir was 10.00 ng/mL, with an analytical range of 10.00 to 10 000.00 ng/mL.

Tenofovir (m/z 288.200/176.200) was extracted using an automated solid phase extraction using tenofovir‐d6 as internal standard (m/z 294.100/182.300). The extracted samples were assessed on a system equipped with an Atlantis dC18, 75 × 4.6 mm, 3 μm column (Waters Corp., Milford, Massachusetts) with water/acetonitrile with ammonium formate and formic acid mobile phase. Over the calibration range, intraday precision and accuracy ranged from 0.79% to 3.40% CV and 97.69% to 99.18%, respectively. Interday precision and accuracy ranged from 1.06% to 3.22% CV and 98.35% to 102.38%, respectively. The lower limit of quantitation for tenofovir was 0.50 ng/mL, with an analytical range of 0.50 to 500.00 ng/mL.

#### Safety and Tolerability

Safety and tolerability were monitored by repeated clinical and laboratory evaluations for the duration of the trial. Safety evaluations included adverse event (AE) reports, laboratory tests, 12‐lead electrocardiograms, and vital signs.

### Statistical Analyses

#### Pharmacokinetics

Plasma islatravir, dolutegravir, and tenofovir pharmacokinetics were summarized descriptively. Pharmacokinetic parameter values were calculated using the software Phoenix WinNonlin version 6.3 (Certara, Princeton, New Jersey). C_max_, C_24_, and t_max_ values were obtained directly from the observed plasma concentration–time data. AUC_0‐inf_ and AUC_0‐24_ were calculated using the linear trapezoidal method for ascending concentrations and the log trapezoidal method for descending concentrations (linear‐up/log‐down). The apparent terminal half‐life was calculated as the quotient of the natural log(ln) of 2 and λz (ln[2]/λz), where λz was the apparent first‐order terminal elimination rate constant calculated from the slope of the linear regression of the terminal log‐linear portion of the plasma concentration–time profile.

Individual AUC_0‐inf_ and C_max_ values for plasma islatravir were natural log‐transformed before analysis and evaluated separately using a linear mixed‐effects model with a fixed‐effect term for treatment. An unstructured covariance matrix was used to allow for unequal treatment variances and to model the correlation between the treatment measurements within each participant. Kenward and Roger's method was used to calculate the denominator degrees of freedom for the fixed effects. Two‐sided 90% confidence intervals (CIs) were constructed for the difference in least‐squares means on the log scale for AUC_0‐inf_ and C_max_. Exponentiating the 90%CI on the log scale, 90%CIs for the geometric least‐squares mean ratios (islatravir plus dolutegravir plus TDF/islatravir alone) were provided for the AUC_0‐inf_ and C_max_.

Individual AUC_0‐24_, C_max_, and C_24_ of dolutegravir and tenofovir were natural log‐transformed and analyzed separately using the same model described for islatravir analysis, with the point estimates and the corresponding 90%CIs for geometric least‐squares mean ratios provided.

#### Safety and Tolerability

Incidence of adverse experiences was descriptively summarized.

## Results

### Participants

A total of 12 participants were enrolled in and completed both periods of the trial. Participant demographics are summarized in Table 1.

**Table 1:** Demographics of Study Participants

Characteristic	Study Participants (N = 12)
Sex, n (%)	
Male	5 (42)
Female	7 (58)
Age, y	
Mean (SD)	39.2 (8.1)
Median (range)	39.0 (25‐53)
Weight, kg	
Mean (range)	71.7 (58‐88)
BMI, kg/m^2^	
Mean (range)	26.7 (21‐31)
Race, n (%)	
Black or African American, Asian	1 (8.3)
White	11 (91.7)
Ethnicity, n (%)	
Hispanic or Latino	11 (91.7)
Not Hispanic or Latino	1 (8.3)

BMI, body mass index; SD, standard deviation.

### Pharmacokinetics

#### Plasma Pharmacokinetic Analysis for Islatravir

**Figure 2:**
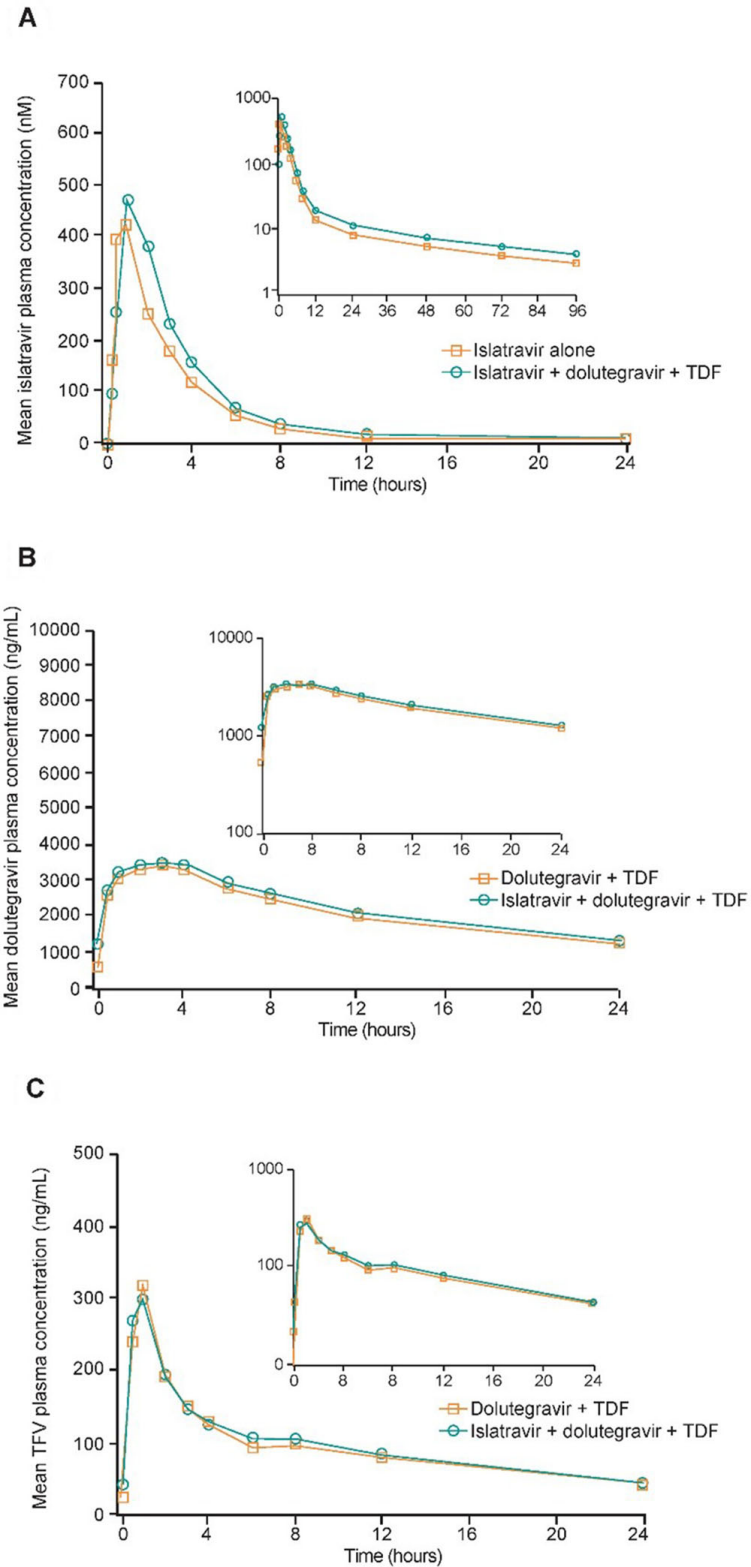
Arithmetic mean plasma concentration–time profiles. (A) Islatravir: following single‐dose administration of 20 mg islatravir with and without multiple doses of 50 mg dolutegravir and 300 mg TDF administered once daily for 8 days to men and women without HIV (N = 12). Inset, semilog scale; 1 nM of islatravir is equivalent to 0.293 ng/mL. (B) Dolutegravir: following multiple‐dose administration of 50 mg dolutegravir and 300 mg TDF once daily dosed to steady state (7 days) with and without a single dose of 20 mg islatravir to men and women without HIV (N = 12). Inset, semilog scale. (C) Tenofovir: following multiple‐dose administration of 50 mg dolutegravir and 300 mg TDF once daily dosed to steady state (7 days) with and without a single dose of 20 mg islatravir to men and women without HIV (N = 12). Inset, semilog scale. TDF, tenofovir disoproxil fumarate; TFV, tenofovir.

Coadministration of islatravir with steady‐state dolutegravir and TDF increased islatravir AUC_0‐inf_ by 28% with comparable C_max_, t_max_, and apparent terminal t_1/2_ values compared with islatravir administered alone (Table 2 and Figure 2A).

**Table 2:** Summary of Plasma Pharmacokinetics of Islatravir After a Single Dose of 20 mg Islatravir With or Without Coadministration of Multiple Doses of 50 mg Dolutegravir and 300 mg TDF to Men and Women Without HIV

	Islatravir Alone^a^		Islatravir + Dolutegravir + TDF^b^		Islatravir + Dolutegravir + TDF / Islatravir Alone		
	N = 12		N = 12				
Pharmacokinetic Parameter	AM (SD)	GM (95%CI)	AM (SD)	GM (95%CI)	AMR (SD)	GMR (90%CI)	Within‐Participant CV, %^c^
AUC_0‐inf_, μM × h^d^, ^g^	2.00 (0.353)	1.97 (1.77‐2.19)	2.53 (0.319)	2.51 (2.31‐2.74)	1.29 (0.17)	1.28 (1.19‐1.37)	9.8
C_max_, μM^d^, ^g^	0.495 (0.135)	0.479 (0.403‐0.568)	0.522 (0.114)	0.510 (0.438‐0.593)	1.10 (0.30)	1.07 (0.93‐1.22)	18.6
t_max_, h^e^	0.75 (0.50‐3.01)		1.00 (0.50‐2.01)				
Apparent terminal t_1/2_, h^f^	57.2 (8.4)	56.6 (15.1)	56.2 (5.8)	55.9 (10.4)			

AM, arithmetic mean; AMR, arithmetic mean ratio; AUC_0‐inf_, area under the plasma concentration–time curve from time 0 to infinity; CI, confidence interval; C_max_, maximum observed plasma concentration; CV, coefficient of variation; GM, geometric least‐squares mean; GMR, geometric least‐squares mean ratio; SD, standard deviation; t_1/2_, half‐life; TDF, tenofovir disoproxil fumarate; t_max_, time to reach maximum observed plasma concentration.

^a^ Single dose of 20 mg islatravir administered on day 1 in period 1.

^b^ Multiple oral once‐daily doses of 50 mg dolutegravir and 300 mg TDF coadministered on days 1 through 11 with a single oral dose of 20 mg islatravir on day 8 in period 2.

^c^ Within‐participant CV (%) estimated based on the elements of the variance‐covariance matrix: CV (%) = 100 × sqrt[(s^2^_A_ + s^2^_B_ ‐2 × s_AB_)/2].

^d^ Back‐transformed least‐squares means and CIs from linear mixed‐effects model performed on natural log‐transformed values.

^e^ Median (min‐max) reported for t_max_.

^f^ Geometric mean and geometric CV (%) is reported for apparent terminal t_1/2_.

^g^ 1 μM of islatravir is equivalent to 293 ng/mL.

#### Plasma Pharmacokinetic Analysis for Dolutegravir and Tenofovir

Coadministration of multiple once‐daily oral doses of dolutegravir and TDF with a single oral dose of islatravir resulted in comparable steady‐state dolutegravir (Table 3 and Figure 2B) and tenofovir (Table 4 and Figure 2C) pharmacokinetics compared with administration without islatravir.

**Table 3:** Summary of Plasma Pharmacokinetics of Dolutegravir After Administration of Multiple Oral Daily Doses of 50 mg Dolutegravir and 300 mg TDF With and Without a Single Oral Dose of 20 mg Islatravir to Men and Women Without HIV

	Dolutegravir + TDF^a^		Islatravir + Dolutegravir + TDF^b^		Islatravir + Dolutegravir + TDF / Dolutegravir + TDF		
	N = 12		N = 12				
Pharmacokinetic Parameter	AM (SD)	GM (95%CI)	AM (SD)	GM (95%CI)	AMR (SD)	GMR (90%CI)	Within‐Participant CV, %^c^
AUC_0‐24_, ng × h/mL^d^	51 200 (19 100)	48 000 (37 900‐60 900)	54 300 (16 900)	52 000 (42 700‐63 200)	1.09 (0.12)	1.08 (1.02‐1.14)	7.5
C_max_, ng/mL^d^	3720 (1350)	3500 (2770‐4430)	3720 (1080)	3570 (2950‐4330)	1.03 (0.17)	1.02 (0.94‐1.11)	11.5
C_24_, ng/mL^d^	1220 (502)	1120 (861‐1470)	1310 (476)	1240 (990‐1550)	1.11 (0.14)	1.10 (1.03‐1.17)	8.6
t_max_, h^e^	2.50 (0.50‐4.13)		3.00 (1.00‐6.03)				
Apparent terminal t_1/2,_ h^f^	15.3 (2.1)	15.2 (14.0)	15.7 (2.7)	15.5 (16.2)			

AM, arithmetic mean; AMR, arithmetic mean ratio; AUC_0‐24_, area under the plasma concentration–time curve over the last 24‐hour dosing interval; C_24_, plasma concentration at 24 hours after dosing; CI, confidence interval; C_max_, maximum observed plasma concentration; CV, coefficient of variation; GM, geometric least‐squares mean; GMR, geometric least‐squares mean ratio; SD, standard deviation; t_1/2_, half‐life; TDF, tenofovir disoproxil fumarate; T_max_, time to reach maximum observed plasma concentration.

^a^ Multiple oral once‐daily doses of 50 mg dolutegravir and 300 mg TDF administered on days 1 through 7 in period 2.

^b^ Multiple oral once‐daily doses of 50 mg dolutegravir and 300 mg TDF administered on days 1 through 11 coadministered with a single oral dose of 20 mg islatravir on day 8 in period 2.

^c^ Within‐participant CV (%) estimated on the basis of the elements of the variance‐covariance matrix: CV (%) = 100 × sqrt[(s^2^_A_ + s^2^_B_ ‐2 × s_AB_)/2].

^d^ Back‐transformed least‐squares means and CIs from linear mixed‐effects model performed on natural log‐transformed values.

^e^ Median (min‐max) reported for t_max_.

^f^ Geometric mean and geometric CV (%) is reported for apparent terminal t_1/2_.

**Table 4:** Summary of Plasma Pharmacokinetics of Tenofovir After Administration of Multiple Oral Daily Doses of 50 mg Dolutegravir and 300 mg TDF With and Without a Single Oral Dose of 20 mg Islatravir to Men and Women Without HIV

	Dolutegravir + TDF^a^		Islatravir + Dolutegravir + TDF^b^		Islatravir + Dolutegravir + TDF / Dolutegravir + TDF		
	N = 12		N = 12				
Pharmacokinetic Parameter	AM (SD)	GM (95%CI)	AM (SD)	GM (95%CI)	AMR (SD)	GMR (90%CI)	Within‐Participant CV, %^c^
AUC_0‐24_, ng × h/mL^d^	2250 (377)	2220 (1990‐2480)	2350 (332)	2330 (2110‐2570)	1.06 (0.19)	1.05 (0.96‐1.14)	11.4
C_max_, ng/mL^d^	346 (65.9)	339 (297‐387)	343 (83.8)	333 (282‐393)	1.00 (0.23)	0.98 (0.88‐1.10)	15.5
C_24_, ng/mL^d^	43.6 (9.53)	42.6 (37.3‐48.8)	45.3 (7.63)	44.8 (40.3‐49.7)	1.06 (0.17)	1.05 (0.97‐1.14)	10.9
t_max_, h^e^	1.00 (0.50‐1.01)		1.01 (0.51‐2.01)				
Apparent terminal t_1/2_, h^f^	13.8 (1.6)	13.7 (12.2)	13.3 (1.4)	13.2 (11.0)			

AM, arithmetic mean; AMR, arithmetic mean ratio; AUC_0‐24_, area under the plasma concentration–time curve over the last 24‐hour dosing interval; C_24_, plasma concentration at 24 hours after dosing; CI, confidence interval; C_max_, maximum observed plasma concentration; CV, coefficient of variation; GM, geometric least‐squares mean; GMR, geometric least‐squares mean ratio; SD, standard deviation; t_1/2_, half‐life; TDF, tenofovir disoproxil fumarate; t_max_, time to reach maximum observed plasma concentration.

^a^ Multiple oral once‐daily doses of 50 mg dolutegravir and 300 mg TDF on days 1 through 7 in period 2.

^b^ Multiple oral once‐daily doses of 50 mg dolutegravir and 300 mg TDF on days 1 through 11 coadministered with a single oral dose of 20 mg islatravir on day 8 in period 2.

^c^ Within‐participant CV (%) estimated based on the elements of the variance‐covariance matrix: CV (%) = 100 × sqrt[(s^2^_A_ + s^2^_B_ ‐2 × s_AB_)/2].

^d^ Back‐transformed least‐squares means and CIs from linear mixed‐effects model performed on natural log‐transformed values.

^e^ Median (min‐max) reported for t_max_.

^f^ Geometric mean and geometric CV (%) is reported for apparent terminal t_1/2_.

### Safety and Tolerability

Administration of islatravir alone and in combination with dolutegravir and TDF was generally well tolerated. In total, 6 of 12 participants reported AEs, of whom 5 reported AEs considered to be drug‐related: 3 participants after administration of islatravir, 3 participants after administration of dolutegravir and TDF, and 1 participant after coadministration of all 3 therapies. All AEs were mild or moderate in intensity and resolved by the end of the study.

The most common drug‐related AE was headache, reported by 3 participants, while all other drug‐related AEs—abdominal discomfort, constipation, nausea, chest discomfort, viral infection, back pain, dysgeusia, and alopecia—were each reported by 1 participant.

No deaths or serious AEs occurred, and no participants discontinued because of an AE. No clinically meaningful treatment‐related changes were observed in laboratory, vital signs, or electrocardiogram safety parameter values.

## Discussion

Islatravir is a novel NRTTI that has shown potential to be efficacious in the treatment and prevention of HIV‐1 infection.^8^, ^13^, ^17^, ^24^ This trial was conducted to investigate the potential impact of coadministration of islatravir with dolutegravir and TDF on the pharmacokinetic and safety profile of these agents. The trial results demonstrated no clinically meaningful pharmacokinetic drug‐drug interactions, with no safety issues identified with coadministration.

New treatment options are needed to meet the diverse needs of people living with HIV, including heavily treatment‐experienced individuals who have exhausted all or nearly all antiretroviral therapies as a result of extensive multidrug resistance or intolerance.^3^, ^25^ Islatravir has the potential to be the first NRTTI for the treatment and prevention of HIV‐1 infection. It is essential, therefore, to fully establish the pharmacologic and clinical profile of islatravir, including when islatravir is used in combination with other antiretrovirals, to meet such needs. The combination of islatravir and doravirine is currently being investigated not only in treatment‐naive and treatment‐experienced individuals but also in heavily treatment‐experienced people living with HIV‐1. Preclinical studies have demonstrated that islatravir has high potency against HIV‐1 and NRTI‐resistant variants, with the potential to have a high barrier to the development of resistance,^26^ while doravirine has been shown to be effective against common nonnucleoside reverse transcriptase inhibitor–resistant HIV‐1 variants.^27^, ^28^ The favorable pharmacokinetics and drug‐interaction profiles of islatravir coadministered with doravirine,^29^ doravirine coadministered with dolutegravir,^30^ and doravirine coadministered with TDF,^31^ and the similar pharmacokinetics of islatravir in individuals living with and without HIV‐1^13^ have already been established. The findings demonstrated in this study add support to the further investigation of adding a combination of islatravir and doravirine to optimized background antiretroviral therapy comprising dolutegravir and TDF in heavily treatment‐experienced people living with HIV‐1.

The pharmacokinetic findings of this study were expected based on the known metabolic pathways of each antiretroviral agent. Dolutegravir is hepatically metabolized by uridine diphosphate glucuronosyltransferases and CYP3A4 and does not induce or inhibit CYP enzymes or uridine diphosphate glucuronosyltransferase 1A1 at physiologic concentrations.^15^, ^32^ TDF is metabolized by plasma and tissue esterases to tenofovir, which is then phosphorylated intracellularly to the active form, tenofovir diphosphate. Tenofovir is not metabolized by CYP enzymes, nor does it induce or inhibit these enzymes. Tenofovir is renally eliminated as unchanged drug by glomerular filtration and active tubular secretion.^33^ Islatravir is eliminated enzymatically primarily by adenosine deaminase–mediated metabolism^34^ and is not expected to have significant drug‐drug interactions. No clinically meaningful drug‐drug interactions were demonstrated following coadministration of islatravir with doravirine.^29^ Furthermore, no significant drug‐drug interactions have been observed following coadministration of islatravir with levonorgestrel and/or ethinyl estradiol, common components of hormonal contraceptives.^35^

A potential limitation of this trial is that only a single dose of islatravir was administered during each treatment period. However, given what is known about islatravir, dolutegravir, and tenofovir, islatravir is not anticipated to affect the pharmacokinetics of either drug regardless of dose or number of doses. Dolutegravir and TDF were administered in multiple doses to achieve near‐maximum induction potential and were dosed for an additional 3 days after islatravir dosing to maintain enzyme inhibition through the islatravir pharmacokinetic collection period. Another limitation of the trial is the use of a fixed‐sequence vs randomized crossover design selection, which may have led to a period effect. Owing to the operational complexity of the crossover design, the fixed‐sequence approach was selected, with the expectation that a period effect would not significantly impact the results.

## Conclusions

The coadministration of islatravir with dolutegravir and TDF revealed no clinically meaningful impact on islatravir, dolutegravir, and tenofovir pharmacokinetics, with no safety issues identified. These data support continued clinical investigation of islatravir as an option for the treatment and prevention of HIV‐1 infection.

## Conflicts of Interest

D.J.R., S.Z., K.L.F., S.F.B., R.P.M., E.F., S.A.S., and M.I. are current or former employees of Merck Sharp & Dohme Corp., a subsidiary of Merck & Co., Inc., Kenilworth, New Jersey, and may have stock options in Merck & Co., Inc.

## Funding

Funding for this research was provided by Merck Sharp & Dohme Corp., a subsidiary of Merck & Co., Inc., Kenilworth, New Jersey (MSD).

## Data‐Sharing Statement

The data‐sharing policy for Merck Sharp & Dohme Corp., a subsidiary of Merck & Co., Inc., Kenilworth, New Jersey, including restrictions, is available at http://engagezone.msd.com/ds documentation.php. Requests for access to the clinical study data can be submitted through the EngageZone site or via email to dataaccess@merck.com .
